# Betahistine Treatment in a Cat Model of Vestibular Pathology: Pharmacokinetic and Pharmacodynamic Approaches

**DOI:** 10.3389/fneur.2018.00431

**Published:** 2018-06-11

**Authors:** Brahim Tighilet, Jacques Léonard, Isabelle Watabe, Laurence Bernard-Demanze, Michel Lacour

**Affiliations:** ^1^Aix-Marseille Université – Centre National de la Recherche Scientifique, Laboratoire de Neurosciences Sensorielles et Cognitives, UMR 7260, Physiopathologie et Thérapie des Désordres Vestibulaires, Centre Saint-Charles, Marseille, France; ^2^Service ORL et de Chirurgie Cervico-Faciale Hôpital de la Conception Marseille, Marseille, France

**Keywords:** unilateral vestibular neurectomy, posture recovery, betahistine, monoamine oxydase inhibitor, histamine, cat

## Abstract

This study is a pharmacokinetic (PK) and pharmacodynamics (PD) approach using betahistine doses levels in unilateral vestibular neurectomized cats (UVN) comparable to those used in humans for treating patients with Menière's disease. The aim is to investigate for the first time oral betahistine administration (0.2 and 2 mg/kg/day) with plasma concentrations of betahistine and its major metabolite 2-pyridylacetic acid (2-PAA) (*N* = 9 cats), the time course of posture recovery (*N* = 13 cats), and the regulation of the enzyme synthesizing histamine (histidine decarboxylase: HDC) in the tuberomammillary nuclei (TMN) of UVN treated animals (*N* = the same 13 cats plus 4 negative control cats). In addition the effect of co-administration of the lower betahistine dose (0.2 mg/kg/day) and selegiline (1 mg/kg/day), an inhibitor of the monamine oxidase B (MAOBi) implicated in betahistine catabolism was investigated. The PK parameters were the peak concentration (*C*_max_), the time when the maximum concentration is reached (*T*_max_) for both betahistine and 2-PAA and the area under the curve (AUC). The PD approach consisted at quantifying the surface support area, which is a good estimation of posture recovery. The plasma concentration-time-profiles of betahistine and 2-PAA in cats were characterized by early *C*_max_-values followed by a phase of rapid decrease of plasma concentrations and a final long lasting low level of plasma concentrations. Co administration of selegiline and betahistine increased values of *C*_max_ and AUC up to 146- and 180-fold, respectively. The lowest dose of betahistine (0.2 mg/kg) has no effects on postural function recovery but induced an acute symptomatic effect characterized by a fast balance improvement (4–6 days). The higher dose (2 mg/kg) and the co-administration treatment induced both this acute effect plus a significant acceleration of the recovery process. The histaminergic activity of the neurons in the TMN was significantly increased under treatment with the 2 mg/kg betahistine daily dose, but not with the lower dose alone or in combination with selegiline. The results show for the first time that faster balance recovery in UVN treated cats is accompanied with high plasma concentrations of betahistine and 2-PAA, and upregulation of HDC immunopositive neurons in the TMN. The higher betahistine dose gives results similar to those obtained with the lower dose when co-administrated with an inhibitor of betahistine metabolism, selegiline. From a clinical point of view, the study provides new perspectives for Menière's disease treatment, regarding the daily betahistine dose that should be necessary for fast and slow metabolizers.

## Introduction

Static and dynamic deficits are observed in most of the species at rest and while moving head and body in space, respectively, after peripheral labyrinth lesion on one side. The spontaneous vestibular nystagmus on one hand, the head tilt, circling, falls to the lesion side, increased support surface on the other hand, constitute the main signs for the static oculomotor and postural syndrome. The asymmetrical resting discharge of the vestibular nuclei (VN) cells, with decreased electrical activity on the lesion side and near normal activity on the intact side, is responsible for the static deficits (1–7). Oscillopsia and blurred vision occur when vestibular patients move fastly their head in space to the disease side, as the result of decreased gains of the vestibulo-ocular reflexes. This major dynamic oculomotor deficit is responsible for both gaze instabilities and falls. In addition, impairment of the vestibulo-spinal reflexes contribute to accentuate the balance problems during stance and gait. As a rule, there is a spontaneous recovery of the vestibular deficits, referred to as “vestibular compensation” in the literature, which shows rather complete or poor compensation for the static and dynamic deficits, respectively (6, 8–11). The functional recovery includes plastic events within the VN, rebalancing the spontaneous neuronal activity on both sides, responsible for the static deficits compensation. The dynamic functions are recovered by means of sensory and behavioral substitution processes, remodeling and changes in the brain functional connectivity.

Neuropharmacology of the vestibular system is relatively well-documented (12), and many experiments showed vestibular compensation improvement with various drugs in different animal models (8, 13–15). Agonists and antagonists of neurotransmitters (Histamine, GABA) and calcium channels antagonists have been found to play a significant role in the recovery process (12, 15–20).

Histamine receptors (HR) modulators are historically among the first anti-vertigo drugs used (15, 18). There is a strong rationale for the use of this drug class: first, the presence of HR and innervation in central vestibular relays, second, the demonstration of the neuromodulatory role of histamine in the VN; and third, the dose-dependent effect of HR ligands on the vestibular compensation (21–25). However, the relation between biological activity and exposure levels of betahistine in unilateral vestibular neurectomized (UVN) cats is unknown. In addition, betahistine efficacy in vestibular loss patients is still controversial although it was found to improve the recovery of the static symptoms in Menière's disease patients after curative unilateral vestibular neurectomy (26). Most of the clinical trials were not performed, however, in gold standard conditions (double-blind, randomized, placebo controlled), leading to unconclusive data [(27), for recent meta-analyses and reviews]. In addition, the efficient daily dose of betahistine to be administrated in patients remains still controversial. Dose-dependent effects have been demonstrated in animal models on both improvement of the cochleo-vestibular blood flow (28–31) and acceleration of the recovery time-course of posture and balance (21, 25). The daily doses recommended (48 mg/kg) seem not efficient in Menière's disease patients (32) while higher dosages reduce significantly more the number of vertigo attacks per month in this pathology (33). The lack of efficacy with usual betahistine doses (16–48 mg/day) could be due to its fast metabolism, given that 95% of the drug is fastly metabolized by the monoamine oxidase a/b. Inhibitors of the monoamine oxidase (selegiline: IMAOb) were found to increase the plasma concentration of betahistine and to decrease the plasma concentration of betahistine metabolites (34). Co-administration of betahistine and selegiline could be a therapeutic way for the future. Another question is the possible role of the betahistine metabolites since it was demonstrated that they had the same targets than betahistine. Indeed, aminoethylpyridine and hydroxyethylpyridine, two betahistine metabolites, were found to induce roughly similar effects on the micro-circulation than betahistine in the guinea pig model (29).

The current study was performed to investigate the pharmacokinetic (PK) and pharmacodynamic (PD) parameters of betahistine at dose levels of 0.2 mg/kg/day and 2 mg/kg/day, that is, at dose levels comparable to those provided daily in Menière's disease patients, and after co-administration of betahistine at the lowest dose (0.2 mg/kg/day) and an inhibitor of the monamine oxidase B (MAOBi) (selegiline: 1 mg/kg/day). The rationale of the study was to investigate the PK/PD relationship for posture recovery, plasma concentration of betahistine and its metabolite 2-pyridylacetic acid (2-PAA). In parallel histamine regulation was investigated by labeling histidine decarboxylase (HDC: histamine synthesizing enzyme) in the posterior hypothalamus (tuberomammillary nuclei: HDC-immunopositive neurons).

## Materials and methods

### Ethics statement

This study was carried out in accordance with the recommendations of the National Institutes of Health Guide for Care and Use of Laboratory Animals (NIH Publication n° 80–23) and the Society for Neuroscience (November 1993). The protocol was approved by the National Ethical Committee (French Agriculture Ministry Authorization: B13-055-25). Male European cats (Isoquimen, Barcelona, Spain) were housed in our animal housing facility (Fédération 3C, Aix-Marseille University) in confortable space (normal diurnal light variations), with free access to food and water. Every attempt was made to minimize both the number and the suffering of animals used in this experiment.

### Study design and drug protocol

#### Vestibular neurectomy

We have previously described the surgery for vestibular neurectomy (35). “Adult male cats weighing 4–5 kg were anesthetized with ketamine (20 mg/kg, i.m.; Rhône Poulenc, Mérieux, France), received an analgesic (Tolfenamic acid: tolfedine, 0.5 ml, i.m.; Vetoquinol, Lure, France) and were kept at physiological body temperature using a blanket. The vestibular nerve was sectioned on the left-side at the post-ganglion level in order to leave the auditory division intact after mastoidectomy, partial destruction of the bony labyrinth, and surgical exposure of the internal auditory canal. Animals were maintained under antibiotics for 7 days and analgesics for 3 days.”

The histological procedures confirming the complete vestibular nerve section have been described previously together with the typical vestibular syndrome (postural, locomotor, and oculomotor) observed immediately after surgery (36). A “strong spontaneous horizontal vestibular nystagmus with its slow phase directed to the lesioned side is observed, associated to a vertical eye deviation. The postural symptom consisted of strong hypotonia of the limb extensors ipsilateral to the lesion and strong hypertonia of the contralateral extensor muscles. The muscular tone asymmetry prevented the animal from holding itself upright and, consequently, all the cats remained lying on their lesioned side after UVN. In addition, a strong head tilt toward the lesioned side associated with a rotation of the chin to the intact side” was seen just after surgery. When the animal is capable to stand erect, it shows an enhanced support surface and falls on its lesioned side. As soon as it can walk, its locomotion trajectory is deviated toward the side of the lesion and it falls many times.

The present study was performed on a total number of 26 cats. Nine UVN cats were used for the PK study, thirteen UVN cats for the behavioral study (including four positive controls without treatment) and for the immunohistochemical study (plus four negative controls without treatment and without vestibular neurectomy).

#### The pharmacokinetic study

The experiments were performed on nine adult male UVN cats randomly allocated into three groups of cats. Three groups of three adult male cats received 0.2 mg/kg betahistine (*N* = 3: cats 1–3), 2 mg/kg betahistine (*N* = 3: cats 4–6), or 0.2 mg/kg betahistine plus 1 mg/kg selegiline (*N* = 3: cats 7–9). Blood samples were collected via jugular venipuncture at pre-dose, 5, 15, 30 min, 1, 2, 3, 4, and 7 h after dosing on day 1 and 21. The samples were frozen at −80°C until analysis. Pharmacokinetic parameters of betahistine and its major metabolite 2-PAA were calculated by applying non-compartmental analysis.

Betahistine and its internal standard, betahistine-d3, were extracted by liquid/liquid extraction from plasma under alkaline conditions toward 1-butanol/isooctane, followed by a second liquid/liquid extraction step of acidic 1-butanol/isooctane toward water. Betahistine concentration in plasma was analyzed using a validated LC-MS/MS method. In brief, an API 4000 (Applied Biosystems) mass spectrometer coupled with a Symbiosis HPLC system (Sparks, Emmen, The Netherlands) was employed to analyze betahistine. Extracts (100 μl) were injected into a XTerra RP18 150 ^*^ 4.6 mm, 3.5 μm column (Waters, Etten-Leur, The Netherlands) and separated at 30°C under isocratic conditions using an aqueous metanol solution containing 0.5 mM ammonium formate as mobile phase. Betahistine was quantified by a tandem mass spectrometer in positive MRM. Parent daughter transition was followed for betahistine (m/z 137 > 94). Betahistine-d3 dihydrochloride was used as internal standard. Pharmacokinetic parameters of betahistine were calculated by applying non-compartmental analysis using the validated PK software WinNonLin, version 5.23 (Pharsight Corporation, Mountain View, CA, USA).

#### Pharmacokinetic evaluation

*C*_max_ and *t*_max_ were derived directly from the plasma concentration-time curves. Linear trapezoidal rule was used to calculate AUC0-t during the part of the curve with increasing plasma concentrations. For the descending part of the curve, the log-linear trapezoidal rule was used. Calculation of the elimination half-life (*t*_1/2_) was evaluated as ln 2/kel, with kel as the slope of the terminal phase of the plasma concentration-time curve.

### Pharmacodynamic study

#### Behavioral investigation

The experiments were performed on 13 adult male European cats randomly allocated into 4 groups of cats.

One positive control group (*N* = 4: cats 10–13) was submitted to a UVN and received post-operatively a vehicle (placebo group). Two groups of cats were treated with betahistine at the lower dose of 0.2 mg/kg/day (*N* = 3: cats 14–16) or at the higher dose of 2 mg/kg/day (*N* = 3: cats 17–19). Another group of cats received the combination of both betahistine at 0.2 mg/kg/day plus selegiline at the dose of 1 mg/kg/day (*N* = 3: cats 20–22).

Placebo treatment was pursued until full recovery of posture control in the control group, that is, 40 days on average. The same post-lesion period was investigated in the three experimental groups receiving betahistine or betahistine + selegiline.

#### Immunohistochemical investigation

Seventeen animals were used for the immunohistochemical study to label the histamine synthesizing enzyme: the HDC. This study was performed on the same four groups of cats used for the behavioral investigation (cats 10–22). These lesioned animals (left UVN) were all killed 40 days after vestibular lesion. A supplementary group of intact animals (*n* = 4: cats 23–26) was used as a negative control group without vestibular lesion.

### Behavioral deficits quantification

#### Static postural deficits

We have described in previous papers how posture deficits and recovery were evaluated (21). The surface delimited by the four legs of the cat was measured while standing erect at rest, without walking. “Support surface is considered a good estimate of postural control since it reflects the cat's behavioral adaptation to compensate the static vestibulospinal deficits induced by the vestibular lesion. As a rule, the surface was very small in the normal cat (about 50–100 cm^2^) and greatly increased in the days following unilateral vestibular lesion. To quantify the support surface, cats were placed in a device with a graduated transparent floor that allowed them to be photographed from underneath. Five repeated measurements were done for each cat tested at each post-operative time, and an average was calculated for each experimental session. The support surface was measured as the surface delimited by the four legs by an image analysis system (Canvas, 9^TM^, Deneba software, Miami, FL). Post-lesion data were compared to pre-lesion values by using individual references, each animal being its own control. Recovery of static posture function was assessed by the changes and development of the support surface over time. The recovery was considered as total when the support surface returned to the pre-operative value (unity, i.e., 1).”

#### Data analysis

Statistical analysis consisted of an ANOVA to test for changes at the different post-lesion days (2, 4, 6, 10, 15, 20, 25, 30, 35, 40 days) for the support surface in each group of cats. Repeated-measure analysis of variance was used to test (1) the effects of betahistine treatment (controls vs. treated cats), (2) the effects of the betahistine dose (0.2 vs. 2 mg/kg), and (3) the effects of additional selegiline on the lowest betahistine dose (0.2 mg/kg betahistine + 1 mg/kg selegiline).

ANOVA was followed by post-*hoc* analysis (Scheffe-test and multicomparison Fisher's test: Statview II software). Results were considered significant at *P* < 0.05. To distinguish between chronic and early effects of betahistine treatment a Two-way-ANOVA with day and treatment as discriminating factors was performed (GraphPad Prism 5.0). The Cohen's *d*-value (two means differences/largest standard deviation) was evaluated to test the size effect.

### Histidine decarboxylase measurement

Tissue preparation, immunohistochemical procedure, and data quantification have been fully described previously (37).

### Tissue preparation

Cats were deeply anesthetized with ketamine dihydrochloride (20 mg/kg, i.m., Rhône Poulenc Mérieux) and perfused under isoflurane intubation (2%), through the ascending aorta, with 1 L of normal saline solution (0.9%) containing 0.1% heparine, and thereafter with 2 L of ice-cold 0.1 M phosphate buffer (PB, pH 7.4) including 4% paraformaldehyde and 0.2% picric acid.

After removal from the skull, the brain was cut into several blocks and post-fixed overnight at 4°C in the same solution. The blocks were then rinsed and cryoprotected by increasing concentrations (10, 20, and 30%) of sucrose solution in 0.1 M PB for 72 h at 4°C. Blocks containing the TMN were rapidly frozen with CO_2_ gas, and coronal sections 20 μm thick were cut in a cryostat (Leica, Rueil-Malmaison, France).

### Immunohistochemical procedure

Free-floating sections were first incubated three times for 5 min in PBS (phosphate buffer saline 0.1 M), then for 1 h in 10% BSA, and 0.25% Triton X-100. Thereafter, sections were incubated under continuous agitation 24 h at 4°C with a Guinea pig polyclonal anti-HDC antiserum (1:1,000 in PBS containing 2% BSA and 0.25% Triton; Fisher scientific). After several rinses (3 × 5 min in PBS with 2% BSA and 3 × 5 min in PBS with 5% BSA) sections were incubated for 1 h, respectively, in biotinylated rabbit anti-guinea pig IgG (1:200 in PBS containing 2% BSA; from Vector Laboratories, Burlingame, CA). After rinses (3 × 5 min in PBS with 2% BSA and 3 × 5 min in PBS with 5% BSA), the sections were processed for immunodetection through the following incubation: 1 h in horseradish peroxidase avidine D (Vector Laboratories, Burlingame, CA), 10 min in a solution containing 0.02% diaminobenzidine, and 5 more minutes in the diaminobenzidine solution with 0.03% hydrogen peroxide added.

### Data quantification

The tuberomammillary nuclei (TMN) of the posterior hypothalamus, the sole structure in the brain containing histaminergic neurons, were identified using Berman's stereotaxic atlas (38). The analysis was performed on the neurons expressing HDC. To produce consistent results of positively immunolabeled tuberomammillary cell bodies, sections were taken from control, lesioned, and treated + lesioned cats. Serial sections in the TMN of each animal were analyzed, five sections at each of the five rostrocaudal levels examined (A12, A11, A10.2, A9.5, and A8.3) being quantified. The number of HDC immunoreactive neurons was quantified by means of a computer-assisted image analysis (DMLB microscope: Leica Microsystems, Wetzlar, Germany) equipped with a DXM 1200 Nikon high-resolution digital camera (1,024 × 1,024 pixels; Nikon, Tokyo, Japan) interfaced to a PC computer employing image software for capturing and processing the images (Lucia G, Nikon, Champigny-sur-Marne, France). Quantification of the immunolabeled neurons was performed via a gray-level method by adjusting a threshold brightness value, and only cells labeled with a gray value above this threshold were taken into account. Reproducibility was assessed by comparing the same data analyzed independently by two researchers blind to the animals' groups. The specific immunolabeling was quantified in each section as the number of labeled neurons, and was automatically computed and evaluated thereafter as the mean (± SEM) for each side, each cat, and each subgroup of cats. To eliminate quantification problems due to potentially asymmetric slides, data were collected from symmetrical slides only, and symmetry was assessed on the basis of the number of cells stained with cresyl violet on each side.

### Statistical analysis

Analysis of variance (ANOVA) was performed to measure the vestibular lesion effect (control vs. UVN cats), side effect (left vs. right, deafferented vs. intact), treatment effect (40 days UVN untreated cats vs. 40 days UVN treated cats) and type of treatment effect (40 days UVN betahistine 2 mg/kg cats vs. 40 days UVN betahistine 0.2 mg/kg cats vs. 40 days UVN betahistine 0.2 mg/kg + selegiline 1 mg/kg cats). The ANOVA was performed on the number of HDC immunoreactive neurons in the TMN of the posterior hypothalamus. Interaction effects were evaluated and post-*hoc* analysis (Scheffe-test and multicomparison Fisher's-test: StatView II, SAS Software Inc., Cary, NC, USA) completed the ANOVA.

## Results

### Pharmacokinetic study

#### Betahistine PK results

The plasma concentration-time profiles of betahistine in cats were characterized by early *C*_max_-values followed by a phase of rapid decrease of plasma concentrations and a final long lasting low level of plasma concentrations. A phase of complete elimination of betahistine from cat plasma was not reached for any dose at the last measured time point of 7 h. An overview of betahistine PKs is presented in Table 1.

**Table 1:** Betahistine PK parameters following administration of different doses of betahistine and co-medication of betahistine with the MAO-B inhibitor selegiline.

**Betahistine/Selegiline**		**Betahistine PK parameters**			
**Doses (mg)**	**Day**	***C*_*max*_ (ng/ml)**	***t*_*max*_ (h)**	AUC (ng*h/ml)	***t*_1/2_ (h)**
0.2/0	1	4.1 ± 2.9	0.25 (0.25)	3.7 ± 2.1	1.9 ± 0.7
	21	0.6 ± 0.4	2 (1; 4)	1.5 ± 0.6	4.0 (*n* = 2)
2.0/0	1	23.8 ± 11.2	0.25 (0.25)	19.7 ± 5.0	3.0 ± 2.6
	21	38.9 ± 34.9	0.5 (0.5)	51.0 ± 42.4	1.6 ± 0.3
0.2/1	1	54.0 ± 58,7	0.25 (0.25)	26.7 ± 24.1	2.5 ± 0.5
	21	13.2 ± 13	1 (0.5; 1)	6.7 ± 5.1	1.9 ± 0.4

The common early *t*_max_ between 0.25 and 1 h, demonstrates a rapid absorption of betahistine from the upper intestinal tract even at high doses of 10 and 50 mg/kg (additional experiments not reported here).

The Table 1 shows that Mean maximal plasma concentrations of betahistine increase after exposure to higher doses (0.2 vs. 2 mg/kg) and also after exposure of the lower dose associated with selegiline (0.2 mg/kg betahistine plus 1 mg/kg selegiline on day 1). Rather similar results were observed on day 21. The AUC parameters showed the same kinetics expression. Mean values for terminal half-lives remained short whatever the treatment condition.

Selegiline co-administration resulted in clear changes of PK parameters. A dose of 1 mg/kg selegiline increased AUC_0−7h_ and *C*_max_-values after a single administration on day 1 ~5-fold. Following repeated administration on day 21 AUC_0−7h_ and *C*_max_-values were 42- and 65-fold higher comparing animals with and without selegiline co-administration.

#### 2-PAA PK results

Plasma concentration-time profiles of 2-PAA showed a high inter-individual variability between individual cats, doses and days (Table 2). Early mean *t*_max_-values for 2-PAA were found between 0.25 and 3 h. Maximal plasma concentrations of 2-PAA in cat plasma were several magnitudes higher compared to betahistine plasma levels, up to 700-fold in the 0.2 mg/kg dose group and up to 77-fold in the 2 mg/kg dose group. A tendency of increased maximal plasma concentrations were detected after repeated administration on day 21. Co-administration of selegiline resulted in strongly reduced *C*_max_-values on both days 1 and 21. Mean AUC_0−10h_-values were also increased with higher betahistine dosage on day 1, and even more enhanced on day 21. Terminal half-lives of 2-PAA were clearly longer (4.5–12.7 h) as for betahistine. The entire data indicate that there is benefit to the addition of the MAO inhibitor selegiline.

**Table 2:** 2-PAA PK parameters following administration of different doses of betahistine and co-medication of betahistine with the MAO-B inhibitor selegiline.

**Betahistine/Selegiline**		**2-PAA PK parameters**			
**Doses (mg)**	**Day**	***C*_*max*_ (ng/ml)**	***t*_*max*_ (h)**	AUC(ng*h/ml)	***t*_1/2_ (h)**
0.2/0	1	346 ± 118	(0.5; 1)	1,522 ± 412	4.5 ± 1.3
	21	422 ± 78	3.0 (3; 7)	2,766 ± 951	12.7 (*n* = 2)
2.0/0	1	1,507 ± 1,201	0.25 (0.25; 1)	9,213 ± 7,416	7.0 ± 0.9
	21	3,007 ± 583	1.0 (0.5; 2)	16,889 ± 6,531	7.2 ± 1.2
0.2/1	1	135 ± 75	(0.25; 3)	765 ± 470	5.3 ± 1.4
	21	276 ± 339	2.0 (1; 4)	2,016 ± 1,948	8.7 (*n* = 2)

### Pharmacodynamic study

#### Postural data: support surface measurements

Repeated-measure analysis of variance showed that group (controls vs. betahistine treated cats: [*F*_(3, 9)_ = 5.879; *P* < 0.016] and post-lesion time [*F*_(9, 81)_ = 33.18; *P* < 0.0001] constituted the main fixed effects causing the variation among cats. A significant interaction group X post-lesion time was also observed [*F*_(27, 81)_ = 3.953; *P* < 0.0001]. Post-*hoc* statistical power analyses performed with Fisher, Scheffe, and Bonferroni tests on the postural deficits data strengthened the significant effects of betahistine on posture recovery. Comparison of the control group with the different betahistine treated groups showed significant effect sizes with Cohen's *d* analysis (Supplementary Material).

The group of cats receiving the lower dose (0.2 mg/kg) did not significantly differed from the controls [*F*_(1, 5)_ = 2.82; *P* < 0.15] with respect to complete time of recovery, which is in both groups around 6 weeks. However, the statistical evaluation pointed to significant differences due to post-lesion time [*F*_(9, 45)_ = 26.864; *P* < 0.0001] and, more interestingly, it showed an interaction post-lesion time/group [*F*_(9, 45)_ = 3.524; *P* < 0.002]. The Bonferroni post-test applied to these two groups showed significant differences at the particular D+4 and D+6 time delays (*p* < 0.01). Therefore, this significant improvement of the low dose group compared to untreated cats was only relevant for the first week of treatment but not for the entire period until recovery.

The two groups treated with the higher betahistine dose (2 mg/kg) or with the lower betahistine dose + selegiline (0.2 + 1 mg/kg) differed significantly from the controls [*F*_(1, 5)_ = 9.259; *P* < 0.026] and [*F*_(1, 5)_ = 9.479; *P* < 0.027] with respect to complete time of recovery. In these groups, the complete recovery of posture is seen earlier than in the controls: at D+10 in the 2 mg/kg betahistine group, and at D+20 in the 0.2 mg/kg betahistine + 1 mg/kg selegiline. Statistical evaluation pointed also to significant differences due to post-lesion time [*F*_(9, 45)_ = 21.847; *P* < 0.0001 and *F*_(9, 45)_ = 32.361; *P* < 0.0001] and to the interaction post-lesion time/group [*F*_(9, 45)_ = 7.184; *P* < 0.0001 and *F*_(9, 45)_ = 8.484; *P* < 0.0001] for these two groups, respectively.

As with the low dose group using the Bonferroni post-test, significant differences compared to untreated cats were seen during the first week of treatment (D+2, D+4, D+6) and for the entire period until recovery.

**Figure 1:**
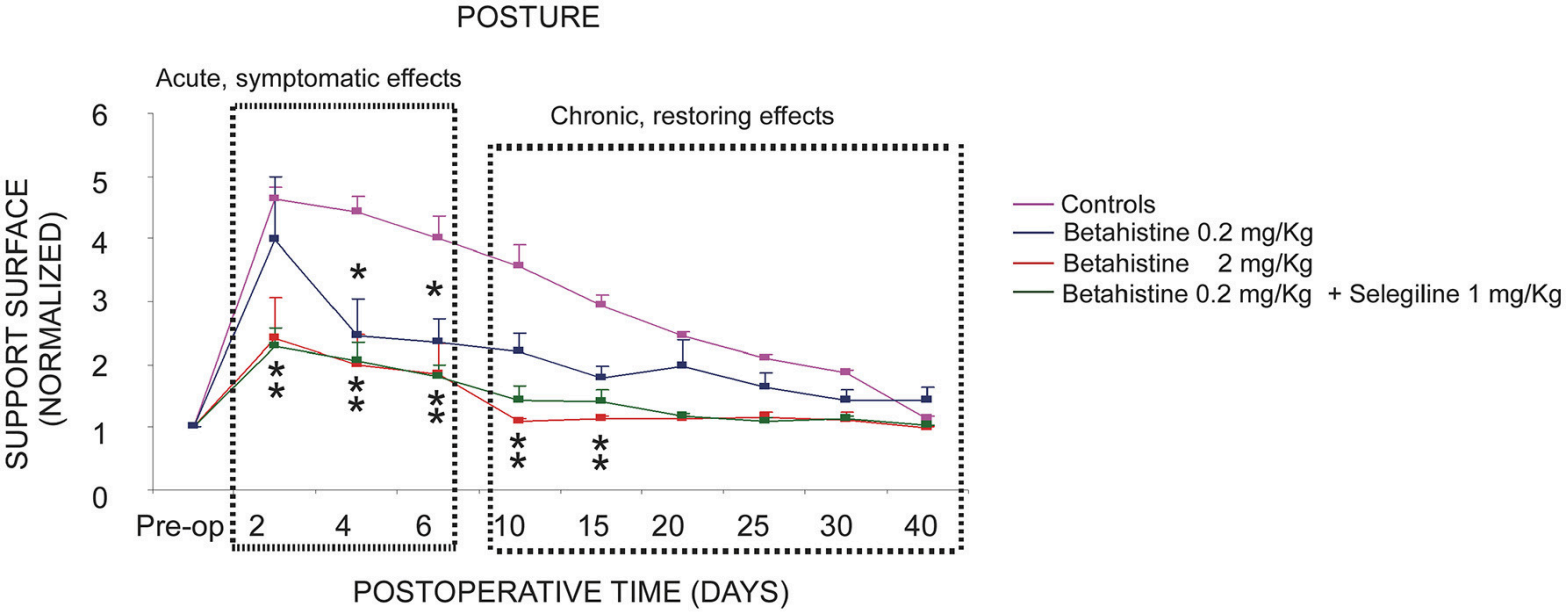
Comparison of the effects of the three oral pharmacological treatments with respect to the controls on the compensation of the static postural deficits. Curves indicating the mean post-operative recovery of the support surface in the four experimental groups of cats: UVN untreated cats (controls, saline water), UVN cats receiving betahistine 0.2 mg/kg *per os*, UVN cats receiving betahistine 0.2 mg/kg plus selegiline 1 mg/kg *per os*, and the UVN group of cats receiving betahistine 2 mg/kg *per os*. Data recorded after vestibular deafferentation were related to individual references and normalized with respect to the pre-operative values referred to unity (one being close to 50 cm^2^). Error bars represent S.E.M. Note the acute and chronic effects of high and low dose betahistine treatment in UVN cats. The normalized effects on posture recovery are roughly similar with the lower dose of betahistine (0.2 mg/kg), the lower dose co administrated with selegiline (1 mg/kg), and the higher dose (2 mg/kg) during this early time window (2–6 days). By contrast, only the higher dose and the co administration treatment induced a significant reduction of the recovery time of balance function during the chronic stage (10–30 days).

In summary, the comparison of the controls with the three experimental groups receiving a post-operative treatment with the low 0.2 mg/kg dose of betahistine, the low 0.2 mg/kg betahistine dose plus the 1 mg/kg dose of selegiline, and the higher 2 mg/kg betahistine dose is shown in the Figure 1. This figure plots the normalized values of the support surface in each group of cats as a function of the post-lesion time. This figure dissociates the symptomatic effects seen during the acute stage from the chronic effects observed later on. The normalized effects on posture recovery are roughly similar with the lower dose of betahistine (0.2 mg/kg), the lower dose co administrated with selegiline (1 mg/kg), and the higher dose (2 mg/kg) during this early time window (2–6 days). By contrast, only the higher dose and the co administration treatment induced a significant reduction of the recovery time of balance function during the chronic stage (10–30 days).

The Table 3 shows the PK/PD relationship evaluated 2 days after the beginning of the different treatments. It clearly indicates that high betahistine *C*_max_- and AUC-values are accompanied with lower normalized values of posture area (*P* < 0.001). Conversely lower betahistine *C*_max_- and AUC-values are accompanied with higher normalized values of posture area (*P* < 0.001).

**Table 3:** PK/PD correlation on day 2 of treatment with different doses of betahistine and co-medication of betahistine with the MAO-B inhibitor selegiline.

**Dose group**	**DAY 2**		
	***C*_*max*_ (pg/ml)**	AUC (h*pg/ml)	**Normalized posture area**
Control	0	0	4.6
BH 0.2 mg/kg	4,105	3,648	3.98
BH 2.0 mg/kg	53,967	26,715	2.4
BH 0.2 mg/kg + Selegiline 1 mg/kg	23,833	19,721	2.3

#### Neurochemical data: HDC protein expression in the TMN

The statistical evaluation shows that all the experimental groups differed significantly from the controls (untreated and unlesioned cats). Among these experimental groups, the UVN D40 + betahistine 2 mg/kg was the only group to differ significantly from the others (UVN D40 + betahistine 0.2 mg/kg, UVN D40 + betahistine 0.2 mg/kg + selegiline 1 mg/kg).

#### Quantification of the HDC immunoreactive neurons in the TMN of the different groups of cats

In the control unlesioned untreated cats, the basal number of HDC immunoreactive neurons was moderate and symmetrical in the bilateral TMN. No significant differences were found in relation with the side (left vs. right) or the cats themselves.

**Figure 2:**
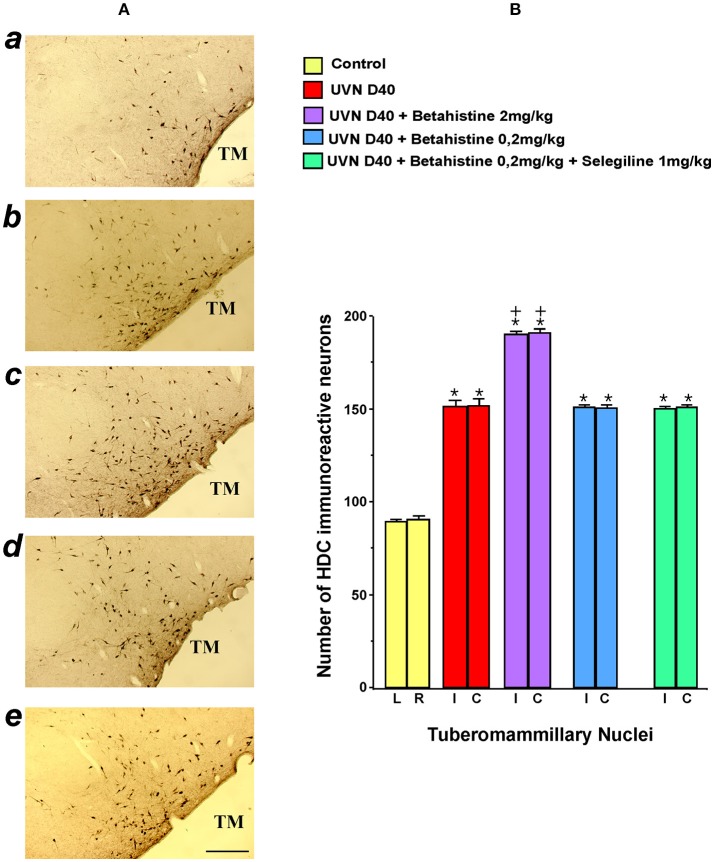
Effects of treatments with different doses of betahistine on the number of histidine decarboxylase immunoreactive neurons in coronal sections of the posterior hypothalamus after unilateral vestibular neurectomy (UVN) in the cat **(A)**. The typical immunolabeling is illustrated from top to bottom in a representative untreated and unlesioned control cat (a), in a UVN cat killed 40 days after unilateral vestibular neurectomy (b), and in three UVN cats treated with 2 mg/kg of betahistine (c), 0.2 mg/kg of betahistine (d), or with a combination of 0.2 mg/kg betahistine and 1 mg/kg of selegiline (e) and killed at the same survival time. Compared to the control, UVN induces a significant increase in the number of histidine immunoreactive neurons in the tuberomammillary nuclei on both sides at this survival time. A strong and significant bilateral increase in the number of histidine immunoreactive neurons is observed exclusively in the tuberomammillary nuclei of UVN cats treated with 2 mg/kg of betahistine compared to the other treated groups. TM: tuberomammillary nucleus; Bar: 1 mm. **(B)**. The quantitative evaluation of the number of histidine immunoreactive neurons in the tuberomammillary nuclei is provided for the controls and the UVN untreated and treated cats (*N* = 3 per group) examined at 40 days survival time, facing the photomicrographs. Data are the mean number of histidine immunoreactive neurons (ordinates). Vertical bars represent the standard errors of the mean. The values are calculated separately for the left and right sides in the control (NS differences) for a direct comparison with the ipsi- and contralateral sides of the other subgroups of animals (UVN D 40 cats, UVN D40 + betahistine 2 mg/kg cats, UVN D40 + betahistine 0.2 mg/kg cats, and UVN D40 + betahistine 0.2 mg/kg + selegiline 1 mg/kg). Note the strong and significant bilateral increase in the number of immunoreactive HDC neurons in the TM of The UVN cats compared with the control (*P* < 0.0001) and of the UVN cats treated with 2 mg/kg of betahistine compared to all the other groups (*P* < 0.0001). + *P* < 0.0001 vs. all the groups; ^*^P < 0.0001 vs. control group.

The data illustrated in the Figure 2 show that vestibular lesion alone (UVN D40 group) induced a significant increase in the number of HDC immunoreactive neurons in the ipsilateral (i: 151.78 ± 3.29) and contralateral (c: 152.4 ± 3.63) TMN compared to the controls (89.15 ± 1.61 and 90.75 ± 1.87, respectively). Additional treatments with betahistine at the low 0.2 mg/kg dose with (150.6 ± 1.43 and 151.6 ± 1.04, respectively) or without 1 mg/kg selegiline (151.3 ± 1.51 and 151.1 ± 1.63, respectively) did not change significantly the number of HDC immunoreactive neurons compared to the UVN D40 group. The only significant increase in the number of HDC immunoreactive neurons was observed bilaterally in the UVN D40 + betahistine 2 mg/kg (190.85 ± 1.58 and 191.68 ± 2.04 in the ipsilateral and contralateral TMN; *p* < 0.0001).

## Discussion

### PK/PD evaluation

The purpose of the PK part of the study was to establish exposure levels of betahistine and its main metabolite 2-PAA in cats at dose levels of 0.2 and 2 mg/kg. The established bio analytical method supported this objective satisfying down to plasma concentrations of 4.51 pg/ml. Nevertheless, PK parameters in cats were with regard to *C*_max_- and AUC-values about 40–50 times higher compared to human. This indicates the unique enzyme system of the feline, and that comparison with doses in humans must be done with caution.

Very early *t*_max_-values for betahistine demonstrated fast absorption processes. And *t*_max_-values of 2-PAA were also found in most cases below 3 h, which documented a fast transformation of the majority of betahistine into 2-PAA, the major metabolite. Moreover, the exposure of 2-PAA which was about 1,000-fold higher than that of betahistine indicated a fast transformation of betahistine to 2-PAA by metabolic processes.

A dose-proportional increase of *C*_max_ and AUC_0−7h_ in the investigated dose range between 0.2 and 2 mg/kg can be assumed although the ratio of dose-normalized *C*_max_- and AUC_0−7h_-values did not match exactly one. The low number of animals per dose group (*n* = 3) and very high inter-individual variability were reasons for this deviation.

Increased values of *C*_max_, AUC_0−7h_ on day 21 compared to day 1 for betahistine and 2-PAA can be attributed to accumulation processes. Based on the available data it cannot be identified which processes caused the increase of *C*_max_ and AUC_0−7h_. Due to the small number of animals per dose group and the high inter-individual variability, this result should be considered with caution and needs confirmation in further studies.

Since early effects of betahistine were observed in all the treated groups of cats while chronic effects were seen only with high dosage or co administration betahistine 0.2 mg/kg plus selegiline 1 mg/kg, it seems that different mechanisms of action could account for the acute vs. chronic effects of betahistine treatment: cochlear blood flow improvement, increase in brain histamine synthesis and release via histamine H_3_ blockade, increase of the betahistine-induced brain arousal level.

Functionally, the acute effect might be related to an action on the vascular tree as seen in experimental models by the betahistine-induced increase in cochlear and vestibular blood flow (28). This mechanism of action has been attributed to a direct action on the histamine H3 heteroreceptors located in the cochlear vascular network (30), and it is supposed to be responsible for the reduction in the number of vertigo attacks in Menière's disease patients. Ihler et al. (28) showed that betahistine exerts a dose dependent effect on cochlear stria vascularis blood flow in guinea pig *in vivo*. This dose-dependent effect could explain the observed acute, so-called symptomatic effects since a certain *C*_max_ of betahistine must be reached to induce an acute effect (cf. Table 1). Using micro-FD Glucose μPET imaging in the rat after chemical or surgical unilateral vestibular lesion, Zwergal et al. (39) showed a double effect of betahistine on the metabolic activity in the VN and the cerebellum, two brain structures involved in vestibular compensation. They observed an early increased metabolism during the first post-lesion days, corroborating the acute effect found in our study, and a longer lasting one found also in the present investigation. Increased metabolic activity in such brain structures during vestibular compensation could favor the rebalancing electrical activity between VN on both sides, a key mechanism for functional recovery (6, 40). The increase in cochlear blood flow demonstrated in the guinea pig model was observed also with aminoethylpyridine and hydroxyethylpyridine, two betahistine metabolites, but not with 2-PAA (29). There is no study investigating the role of 2-PAA in the vestibular compensation process.

The chronic restoring effects are possibly linked to neural networks remodeling and could require a higher exposure (2 mg/kg and higher) of betahistine (Figure 1). Significant acceleration of behavioral recovery was observed in the UVN cats treated with betahistine 2 mg/kg, but not with the low dose (0.2 mg/kg). These data confirm our previous works in cats treated with higher betahistine dosages (21, 25), which displayed a faster acceleration of their posture recovery profile compared to untreated or placebo animals. Dose-dependent effects of betahistine on histamine turnover have already been observed in our UVN cats (24). Interestingly, the lowest dose (0.2 mg/kg) co-administrated with selegiline (1 mg/kg) accelerated posture recovery similarly to the higher 2 mg/kg dose. The data strongly support that betahistine effect is dependent on its plasma bioavailability. Selegiline is an MAO enzyme inhibitor that preferentially inhibits MAO-B at the low doses commonly used. It inhibits the re-uptake of catecholamines and is used in Parkinson's disease to increase and prolong the dopamine plasmatic level. This increased dopamine level would explain also why selegiline enhances wakefulness and motivation. Taken together, co-administration of betahistine and selegiline could be an interesting new pharmacological therapy for Menière's disease patients, regarding the daily betahistine dose that should be necessary, particularly for fast metabolizer patients. In addition, motivation and sensorimotor activity are two crucial factors in the recovery of vestibular functions (11), and selegiline could improve the vestibular compensation process by this secondary action mechanism.

### Immunohistochemical data

#### HDC immuno-positive neurons in the TMN after unilateral vestibular neurectomy

Forty days after unilateral vestibular neurectomy, we observed a strong increase in HDC immune-positive neurons in the TMN on both sides. This result supports our previous investigations in UVN cats pointing to bilateral up-regulation of HDC mRNA in the TMN 3 weeks after the lesion (41, 25). The VN neuronal imbalance reported just after vestibular loss [reviewed in (5)] activates a vestibulo-hypothalamic loop responsible for HDC up-regulation. An hypothesis supported by the hypothalamus increased histamine activity reported after vestibular lesion (23), vestibular caloric (42), or hypergravity (43) stimulations, the lack of any histamine change in bilateral labyrinthectomized animals (43), and the response of hypothalamic neurons to electrical stimulation of the VIII^th^ nerve or the lateral VN (44). The asymmetrical firing rate of the MVN (45) and LVN (3) cells found in acute UVN cats can therefore account for the HDC protein up-regulation in the TMN on the lesioned side.

#### HDC immuno-positive neurons in the TMN after unilateral vestibular neurectomy and betahistine exposure

Our results show that betahistine interferes with histaminergic turnover by increasing the number of HDC immunopositive neurons located in the TMN of the posterior hypothalamus. Compared to the bilateral up-regulation observed 40 days post-lesion in our UVN untreated cats, the combination of lesion and betahistine 2 mg/kg induced a significantly higher number of HDC immunopositive neurons on both sides (210% on average) at the same survival time. Betahistine 0.2 mg/kg combined or not with selegiline had similar effects on HDC immunopositive neurons in the TMN than the vestibular neurectomy alone.

As reported previously (24), “betahistine is a partial histamine H1 receptor agonist and a more potent histamine H3 receptor antagonist. Its interaction with the histaminergic system is now well established. The histamine H3 receptor mediates autoinhibition of brain histamine release and autoregulation of histamine synthesis (46–49). By blocking the histamine H3 autoreceptors, betahistine increases the synthesis and release of histamine in the TMN, as shown also with immunohistochemistry, *in situ* hybridization and binding to histamine H3 receptors methods in our cat model” (23–25, 50). Blockade of the histamine H_3_ autoreceptors releases histamine that could rebalance the VN cells activity on both sides by actions at the post-synaptic histamine H_1_ (51) or H_2_ (52–54) receptors. In addition to this specific mechanism of action, betahistine could improve the microcirculation of the inner ear as well as homeostasis of endolymphatic fluid by acting on H3 and H1 receptors (30, 31, 55).

In the UVN cats treated with the combination of betahistine at the low dosage (0.2 mg/kg) and selegiline, we did not observe so high up regulation of the number of HDC immunopositive neurons as found in the UVN cats treated with betahistine 2 mg/kg alone. The explanation might be that selegiline, which is known to inhibit monoamine oxidase (MAO), an enzyme that catalyzes the oxidation of monoamines such histamine, could preserve neuronal histamine concentration. A higher amount of neuronal histamine would prevent an increased amount of the synthesizing enzyme.

#### Clinical relevance and perspectives

This study shows that betahistine effects depend on its plasma bioavailability. This parameter is linked both to daily dosage and metabolism activity of betahistine. In slow metabolizer animal models (cat) the daily dosage can be low (2 mg/kg) while in human beings, with higher metabolism activity, the daily dosage should be increased (56) and/or given on a longer period. Another possibility in vestibular loss patients could be to associate the drug with selegiline, an inhibitor of the MAOBi implicated in betahistine catabolism. In this case, our data in the cat model show that very low doses of betahistine (0.2 mg/kg) in combination with selegiline (1 mg/kg) are sufficient to significantly improve the posture balance recovery. The route of administration of betahistine could also impact its plasma bioavailability. Changing the route of administration could also be an interesting track given the presence of MAO in the digestive tract. One possibility would be to bypass the catabolism of betahistine at the level of the digestive barrier by administering this drug by the nasal route for example.

## Limitations of the study

The relationship between the restoration of posture and the plasma level of betahistine and its metabolite is highly significant. However, our study was performed with a single dose of selegiline only, and on small groups of cats. It would be necessary to confirm these data on a larger number of animals, and correlation analyses would be required also.

## Author contributions

BT and ML conceived and designed the experiments. BT, JL, IW, and LB-D performed the experiments. JL and LB-D analyzed the data. BT and ML wrote the paper.

### Conflict of interest statement

The authors declare that the research was conducted in the absence of any commercial or financial relationships that could be construed as a potential conflict of interest.

## Abbreviations

UVN: unilateral vestibular neurectomy
HDC: histidine decarboxylase (HDC: enzyme synthesizing histamine)
TMN: tuberomammillary nuclei
MAOBi: an inhibitor of the monamine oxidase B
2-PAA: 2-pyridylacetic acid
*C*_max_: peak concentration
*t*_max_: the time when the maximum concentration reached
AUC: area under curve
VN: vestibular nuclei
MVN: medial vestibular nuclei
LVN: lateral vestibular nuclei
HR: histamine receptors
PK: pharmacokinetic
PD: pharmacodynamics
HPLC: high-performance liquid chromatography
LC-MS/MS: liquid chromatography-mass spectrometry
MRM: multiple reaction monitoring
ANOVA: analysis of variance
PB: phosphate buffer
i.m.: intramuscular
PBS: phosphate buffer saline
BSA: bovine serum albumin
IgG: immunoglobulin G
SEM: error standard of the mean
D: day
H: histamine.
