# Supplementary material for: Betahistine Treatment in a Cat Model of Vestibular Pathology: Pharmacokinetic and Pharmacodynamic Approaches
Source: Front Neurol. 2018 Jun 11;9:431. doi: 10.3389/fneur.2018.00431 (PMC6005348; doi:10.3389/fneur.2018.00431)
Supplement: Supplementary file 1 [file Data_Sheet_1.docx]

| Post lesional delay in days | D+2 | D+4 | D+6 | D+10 | D+15 | D+20 | D+25 | D+30 | D+40 |
| --- | --- | --- | --- | --- | --- | --- | --- | --- | --- |
| Control versus Betahistine (0.2 mg): Cohen’s d | 0.657 | 3.289 | 2.510 | 2.708 | 4.224 | 2.064 | 2.870 | 5.146 | -1.447 |
| Control versus Betahistine (2 mg): Cohen’s d | 2.662 | 3.616 | 2.799 | 5.398 | 7.246 | 13.857 | 8.478 | 6.512 | 3.320 |
| Control versus Betahistine (0.2 mg) + Selegiline (1 mg): Cohen’s d | 4.406 | 4.506 | 3.642 | 4.197 | 4.865 | 13.000 | 10.778 | 12.943 | 3.157 |

Table. Cohen's d values showing the effect size of the different pharmacological treatment conditions
